# Specification and function of hemogenic endothelium during embryogenesis

**DOI:** 10.1007/s00018-016-2134-0

**Published:** 2016-02-05

**Authors:** Emily Gritz, Karen K. Hirschi

**Affiliations:** 1 Departments of Medicine, Genetics and Biomedical Engineering, Yale Cardiovascular Research Center, Vascular Biology and Therapeutics Program, and Yale Stem Cell Center, Yale University School of Medicine, 300 George St., New Haven, CT 06511 USA; 2 Department of Pediatrics, Section of Neonatal-Perinatal Medicine, Yale University School of Medicine, 333 Cedar St., New Haven, CT 06511 USA

**Keywords:** Embryogenesis, Developmental hematopoiesis, Hemogenic endothelium, Endothelial to hematopoietic transition

## Abstract

Hemogenic endothelium is a specialized subset of developing vascular endothelium that acquires hematopoietic potential and can give rise to multilineage hematopoietic stem and progenitor cells during a narrow developmental window in tissues such as the extraembryonic yolk sac and embryonic aorta-gonad-mesonephros. Herein, we review current knowledge about the historical and developmental origins of hemogenic endothelium, the molecular events that govern hemogenic specification of vascular endothelial cells, the generation of multilineage hematopoietic stem and progenitor cells from hemogenic endothelium, and the potential for translational applications of knowledge gained from further study of these processes.

## Introduction

Normal embryogenesis is predicated upon the development of a functioning cardiovascular system early in gestation—disruptions in this lead to vascular, cardiac, hematologic disease, and in extreme cases embryonic lethality. As the primitive vasculature expands throughout early development, it remodels and specializes to fulfill the critical metabolic demands of the tissues it supplies [1]. As part of this remodeling, endothelial cells lining the cardiovascular system subspecialize to arterial, venous, lymphatic, and hemogenic fates via a complex network of intersecting molecular pathways. The mechanisms underlying normal vascular development remain as yet incompletely defined, and a greater understanding of these processes will enhance our understanding of certain pathologic states related to the vascular system and how they might be treated.

Hemogenic endothelium is a small, specialized subset of vascular endothelium that acquires hematopoietic potential and can give rise to multilineage hematopoietic stem and progenitor cells (HSPC) within a narrow developmental window within distinct tissues [1]. Work in the last decade has begun to clarify the complex signals that lead to acquisition of this blood-forming potential. Our understanding of the events that lead to hemogenic specification of endothelial cells from vascular endothelium, as well as the events that lead to generation of HSPC from hemogenic endothelium is still in its infancy. This review will outline current knowledge about the historical and developmental origins of hemogenic endothelium, the molecular events that govern hemogenic specification of vascular endothelial cells, the generation of multilineage HSPC from hemogenic endothelium, and the potential for clinical/translational applications of knowledge gained from further study of these processes.

## Developmental and historical origins of hemogenic endothelium

### Primitive hematopoiesis

Hematopoiesis occurs in two waves during embryonic development—the primitive (precirculation) wave and definitive wave. The first blood and endothelial cells are derived from the extraembryonic mesoderm beginning around embryonic day (E) 7.0 [2]; whether they are derived from a common bipotent progenitor (hemangioblast), or are independently fated, remains a subject of debate, as discussed later in this review. Primitive hematopoiesis is marked by the brief emergence of mostly nucleated erythroid progenitors, as well as macrophage progenitors [3], that are generated in parallel with endothelial cells. These primitive erythroid cells are functionally and morphologically distinct from later erythroid progenitors in that they are large, nucleated, express embryonic globins, and are exclusively detected in the extra-embyonic yolk sac during a narrow (~48 h) window before they disappear [3]. Other precirculation extra-embryonic sources of pro-definitive erythroid and myeloid progenitor cells later emerge between E7.5 and E8.0 and include the allantois [4, 5] and para-aortic splanchnopleura (pSP) [6, 7]. These early cells are of great importance to the developing embryo as they sustain embryonic survival until the definitive hematopoietic system is established, and they form structures referred to as “blood islands” around E7.5 [8], in which the cells in the interior give rise to nucleated erythroid and myeloid cells and those on the periphery become lumenized endothelial cells [1]. Blood islands then coalesce to form vascular channels and a plexus network throughout the yolk sac, which conducts oscillatory plasma flow driven by the developing heart tube [9]. By E8.25, a second wave of erythromyeloid progenitor (EMP) cells are generated which also give rise to tissue macrophages that persist into adulthood [10–12]. Following the onset of plasma flow, the immature progenitor cells join the plasma circulation [3, 13].

### Definitive hematopoiesis

Definitive hematopoiesis is the second phase of blood production beginning with production of erythroid and myeloid progenitors that is then followed by generation of long-term repopulating hematopoietic stem cells (HSC) capable of colonizing hematopoietic organs and giving rise to all differentiated blood lineages [14, 15]. Definitive hematopoiesis coincides with the onset of pulsatile blood flow due to coordinated cardiac contraction beginning around E8.25, as the first wave of extra-embryonic primitive erythroid progenitors previously described decrease in number and the previously described second yolk sac wave of erythroid and myeloid cells are generated and enter the plasma circulation [3, 14, 16]. Generation of hematopoietic progenitors then begins in the placenta between E9.0 and 9.5 [17–20]. In the murine embryo proper, definitive hematopoiesis occurs later, within the aorta-gonad-mesonephros (AGM) region peaking at E10.5 [21–23] and is marked by the generation of both progenitor cells, as well as HSC. These AGM and yolk-sac-derived hematopoietic stem and progenitor cells are thought to migrate to, and colonize other sites of definitive hematopoiesis, most notably the fetal liver by E11.0–E12.0 [24–26] and the bone marrow at E16.5 [27], and contribute to the adult HSC pool. While it is clear that progenitor cells emerge from the yolk sac and placental tissues during definitive hematopoiesis, it remains debated whether or not the placenta and yolk sac actually generate HSC de-novo—although it has been shown that the number of HSC colonizing the fetal liver is greater than what can be accounted for by the AGM alone [26]. This suggests that additional sources of HSC, such as the placenta and yolk sac, may exist and contribute to the HSC pool used by the liver for self-expansion [14, 18, 26, 28]. It also remains unclear whether there exists de-novo generation of HSC within fetal liver, fetal bone marrow or postnatal tissues, or if HSC are  merely replenished via self-renewal of yolk sac- and AGM-derived cells [29].

While it has been demonstrated that the yolk sac and AGM are sites of definitive hematopoiesis, the cellular origins of the HSPC within these tissues is still under investigation, and may be different in distinct species. Nonetheless, it is generally thought that murine definitive HSPC are derived from a small subset of vascular endothelium that acquires hemogenic potential, so-called hemogenic endothelium. Interestingly, hematopoietic cells produced by hemogenic endothelial cells have different functional characteristics depending on their site of origin [29]. That is, yolk sac hemogenic endothelial cells give rise to multilineage hematopoietic progenitors that cannot repopulate neonatal or adult bone marrow long-term in vivo [7], and are therefore referred to as multilineage progenitors. In contrast, AGM-derived hematopoietic cells are capable of rescuing irradiated neonatal and adult recipients [30]; thus, these cells are considered definitive HSC. This raises the possibility that not all hemogenic endothelial cells are created equally, or that their anatomic niche influences their function and progeny, in a manner dependent on the developmental timing of their emergence and/or the cellular and extracellular composition of their microenvironment [29].

### Concept evolution of hemogenic endothelium

To best understand the significance of hemogenic endothelial cells in embryonic hematopoiesis, it is useful to consider the evolution of the concept of their existence and function over the course of the last century. Insights gained during their discovery, identification and characterization have helped intertwine the fields of hematopoietic stem cell biology and vascular biology. The concept of hemogenic endothelium dates back in the scientific literature to the late 19th century. Beginning in 1899, several anatomists observed an association of blood forming cells with developing vascular structures across a wide variety of mammalian species including the bat (1899), chick (1907), rabbit (1909), human (1912), pig (1916), and mongoose (1917) [15]. In 1917, anatomist Florence Sabin directly observed in chick embryo studies that “red blood-corpuscles can be seen to grow from the endothelial lining of blood-vessels” [31]. These blood cells were then noted to bud off from vascular endothelial cells into plasma flowing through primitive vascular plexus of the developing chick.

In the 1930s, Murray re-branded Sabin’s “angioblast” as a “hemangioblast” or a subset of mesenchymally-derived primitive endothelium that transiently acquires blood-forming potential [32]. Over the next several decades, however, this concept of the hemangioblast and the questions surrounding an association between vascular endothelium and blood formation went largely unaddressed. In the 1960s, Moore and Owen suggested that all embryonic hematopoiesis occurred in the extra-embryonic yolk sac, and that intra-embryonic blood in later stages of development was a result of migration of yolk sac derived hematopoietic progenitors [15]. This concept of exclusively extra-embryonic hematopoiesis was refuted by Dieterlen-Lièvre and coworkers in 1975 when they demonstrated intraembryonic aortic hemogenesis in quail-chick embryo grafting experiments and renewed interest in the origin of hematopoietic stem and progenitor cells in the developing embryo [15, 33].

### The hemangioblast

Over the next two decades, several groups corroborated the work of Dieterlen-Lièvre and coworkers by demonstrating the presence of hematopoietic stem and progenitor cells in the ventral aspect of the developing dorsal aorta prior to hepatic colonization, and demonstrated that these cell clusters are necessary for the establishment of definitive hematopoiesis [6, 15, 23, 24, 34, 35]. This confirmed association of hematopoietic cells with vascular endothelium brought into question the developmental relationship between hematopoietic cells and vascular endothelium—were they two independent lineages derived from discrete progenitor cell types or did they have a common mesodermally-derived bipotent cell progenitor, the so-called hemangioblast, that transiently gives rise to vascular endothelial and blood cells? [29]. Indeed, endothelial and hematopoietic lineages have been shown to share many common surface markers and transcription factors that are implicated throughout their differentiation. These include mesodermal markers Brachyury (Bry), bone morphogenic protein 4 (BMP4), and vascular endothelial growth factor 2 (VEGFR2 or Flk-1) [36]; shared markers CD34, VE-cadherin, and CD31; and common transcription factors RUNX1 and GATA binding protein 2 (GATA2) [37], lending credibility to the hemangioblast concept. However, to date, there is a lack of convincing evidence to suggest that such an exclusively bipotent mesodermal progenitor exists [29].

The bipotent hemangioblast has been described as co-expressing mesodermal marker Bry and Flk-1, but Bry+Flk-1+ cells have, in fact, been demonstrated to give rise to cardiac, skeletal, and vascular smooth muscle, as well as endothelial cells and blood cells [38, 39]. Padrón-Barthe and coworkers demonstrated by in vivo clonal analysis of murine embryos that the earliest blood and endothelial lineages do not arise from a primitive streak-derived bipotential precursor, but that these lineages are derived from independent epiblast populations [40]. These results are consistent with previous mesodermal grafting studies that also suggest endothelial and blood cell lineages are independently fated during primitive hematopoiesis [41]. It remains possible that there may exist a bipotent mesodermal progenitor giving rise to exclusively endothelial and blood cells, but this has not been definitively demonstrated to date [29]. A middle ground to the hemangioblast debate was proposed by Lancrin and coworkers, wherein the mesodermally-derived hemangioblast gives rise to an endothelial intermediate (hemogenic endothelium) that then gives rise to HSPC [42].

### Hemogenic endothelium

Despite the uncertainty of the existence of hemangioblasts during primitive hematopoiesis, several landmark studies over the last two decades have provided strong evidence that during definitive hematopoiesis in vertebrates, multipotent HSPC arise directly from transiently specialized vascular endothelium (hemogenic endothelium) within the developing AGM between E10.0 and E11.5, prior to appearance in other intra-embryonic hematopoietic organs [6, 23, 24, 34, 35].

The aforementioned early observations of blood cells budding from vascular endothelium have been corroborated by dye-labeled fate tracing approaches that demonstrate across multiple animal models that dye-tagged vascular endothelium in the dorsal aorta gives rise to dye-tagged circulating blood cells [43, 44]. Subsequent in vivo fate tracing studies in murine embryos has further demonstrated emergence of HSC from endothelium from VE-cad+ cells in the dorsal aorta that go on to colonize intra-embryonic hematopoietic sites including the bone marrow, spleen, and thymus [45].

Hematopoietic stem and/or progenitor cell emergence has also been shown to occur within the vascular plexi within the extra-embryonic yolk sac [13, 46, 47], placenta [18], as well as the vitelline artery, umbilical arteries [21, 48, 49], endocardium [50], and head arteries [51]. Multiple real-time imaging studies demonstrating hematopoietic cell emergence from vascular endothelium have been performed in mice and zebrafish [52–54]. Single cell lineage tracing studies have demonstrated multipotent HSPC with cell surface markers CD31 (endothelial cell marker), CD41 (blood cell marker), c-Kit (stem cell growth factor receptor), and SCA-1 (stem cell antigen, also known as Ly6A) arising directly from the ventral wall of the murine dorsal aortae that can be traced to the fetal liver [53], and ultimately to the adult hematopoietic system in mice [45] and in zebrafish [52, 54].

Several studies have addressed the question of whether vascular-derived hematopoiesis truly originates from endothelial cells and is not mesenchymal in origin. In 2002, de Bruijn and colleagues demonstrated using a Ly6A(SCA-1)-GFP transgenic mouse model that all hematopoietic stem cells within the developing aorta are Ly6A-GFP+ and that these cells localize to the endothelial layer of the dorsal aorta, but not to the underlying mesenchyme [55]. To further support the assertion that HSPC are generated from an endothelial intermediate and not the underlying mesenchyme, it has been demonstrated that key hematopoietic regulators such as the transcription factor RUNX1 are expressed at high levels by endothelial cells in hemogenic vascular sites in vertebrates [56]. North and colleagues further demonstrated that while RUNX1-expressing HSC are found in both endothelial and mesenchymal cell fractions in the mouse embryo, the presence of two functional *Runx1* alleles segregates HSC to the endothelial fraction only [57]. More recently, it has been shown that *Runx1* deletion in VE-cadherin expressing endothelial cells results in loss of intra-aortic hematopoietic cluster formation, again corroborating the link between cells of endothelial origin giving rise to hematopoietic clusters via the action of known hematopoietic mediators such as RUNX1 [58]. Additionally, murine AGM-associated hemogenic endothelial cells give rise to either hematopoietic or endothelial lineages but never both [59] and human pluripotent stem cell-derived arterial endothelial cells and hemogenic endothelial cells can be distinguished on the basis of CD184 and CD73 expression [60].

These studies collectively demonstrate, on a functional as well as morphological level, a strong body of evidence for the existence of hemogenic endothelial cells of exclusively vascular origin that give rise to multilineage HSPC during definitive hematopoiesis. However, much less is known about the mechanisms underlying hemogenic specification and their subsequent generation of HSPC.

## Characterization of hemogenic endothelium

In order to characterize the molecular events underlying hemogenic endothelial cell specification and their subsequent generation of HSPC, it is necessary to delineate the phenotype of hemogenic vs. non-blood forming endothelium, so that they can be effectively isolated and studied. Isolation of these cells proves to be a difficult undertaking as hemogenic endothelium represents a small (~1–3 % of murine yolk sac and AGM endothelial cells) and transient population within hematopoietic tissues [13, 61].

To date, no definitive single marker to distinguish hemogenic from nonhemogenic endothelial cells has been identified. Building upon the body of evidence supporting the very existence of hemogenic endothelium, several groups have used flow cytometry techniques to elucidate the phenotypic identity of hemogenic endothelial cells within the yolk sac and AGM to aid in their isolation and further study. In 1997, Kabrun and colleagues described generation of HSPC from Flk1+ endothelium [62]. Shortly thereafter, Nishikawa and colleagues isolated VE-cad+CD45−Ter119− cells from the yolk sac and caudal half of embryo proper of E9.5 mouse embryos and demonstrated that this cell fraction was capable of giving rise to lymphohematopoietic cells in culture. This study confirmed that the VE-cad+ cell fraction co-expressed vascular markers CD34, CD31, and Flk-1, confirming that the isolated cells were indeed endothelial in origin and confirming both the existence and functional capability of hemogenic endothelium to give rise to blood progenitors [63]. The need for VE-cadherin expression was further corroborated by a subsequent study by Fraser and colleagues that demonstrated long term lyphohematopoietic reconstitution potential of VE-cad+/CD45− cells injected into irradiated neonatal mouse recipients [30]. Hemogenic endothelial cells can also be distinguished from nonhemogenic endothelial cells based on differential activity of a regulatory element of a *KDR* promoter enhancer such that only nonhemogenic endothelial cells activate the enhancer [64]. Other studies have since shown that cells co-expressing CD31, CD34, and Flk-1 from both murine and human yolk sac and AGM have demonstrated lympho- and lymphomyelopoietic potential under culture conditions [1, 63, 65]. In addition, cells co-expressing Flk-1 and VE-cadherin exhibit enhanced colony forming ability when compared to Flk-1+VE-cad− cells [30]; however, VE-cadherin expression was not found to be necessary for the blood-forming ability of yolk sac hemogenic endothelial cells in mice [13] or for transition of hemogenic endothelial cells to HSC in zebrafish and mice [66]. It has been shown as well that hemogenic endothelial cells may actually be divided into functionally distinct populations that either generate erythroid and myeloid progenitor cells or exclusively generate HSC [67].

Alpha4-integrin (α4-integrin) has been identified as a marker that distinguishes hemogenic VE-cad+ cells from nonhemogenic VE-cad+ cells [68]. Live cell time-lapsed imaging of embryonic stem cell-derived Flk1+VE-cad-mesodermal cells co-cultured with OP9 stromal cells demonstrated transformation of VE-cad+DI-acyl LDL+ cells with Claudin5 mediated-tight junctions into sheets of cells with endothelial morphology that gave rise to non-adherent, free-floating round CD45+CD41+ hematopoietic progenitor cells [69]. Loss of endothelial markers and acquisition of hematopoietic markers was shown to proceed in a patterned fashion in this model. More recently, a critical role for Stem cell leukemia (SCL) factor in maintenance of hemogenic competence and prevention of cardiomyocyte programming of primitive vascular endothelial cells has been identified [70]. In other recent studies, single hemogenic and non-hemogenic endothelial cells could be isolated from *Runx1* enhancer-reporter mice, which was beneficial for transcriptional profiling of these cell types [59, 69].

Further phenotypic characterization of hemogenic endothelial cells by our group has revealed that blood-forming activity of both yolk sac and AGM tissues is contained within the Hoechst dye effluxing, or “side population” (SP) fraction of cells [13, 61, 71], which is not surprising given that the SP phenotype is also a characteristic of HSC within adult bone marrow [72]. SP cells within the yolk sac and AGM were further fractionated based on endothelial and hematopoietic cell surface markers, and the phenotype of hemogenic endothelium therein has been defined, on a clonal level, as Flk-1+c-Kit+CD45− SP cells [13, 61, 71]. In addition, the Flk-1+c-Kit+CD45− SP hemogenic endothelial cells were demonstrated to give rise to multilineage HSPC that are Flk1−c-Kit+CD45+ SP cells [13], that can be distinguished from mature blood cell types with a Flk1−CD31±CD45+ non-SP phenotype.

## Specification of hemogenic endothelium

**Fig. 1:**
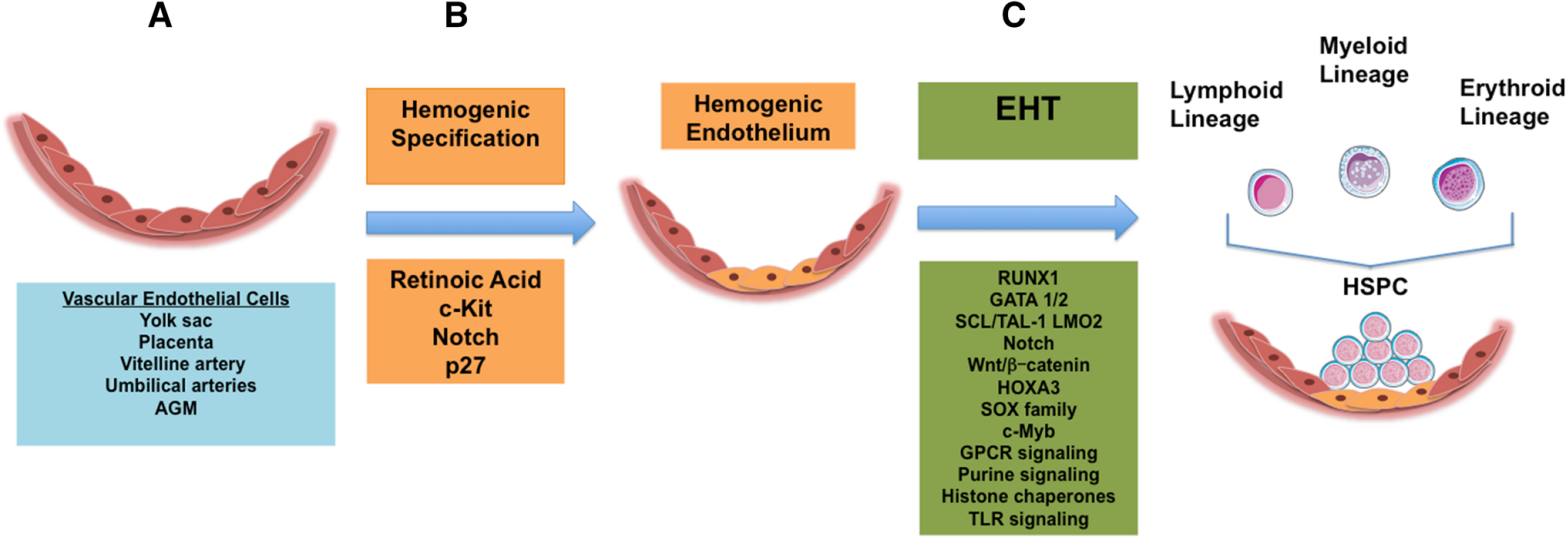
Summary of regulation of primitive hematopoietic specification and generation of hematopoietic stem cells during embryonic development. **a** Schematic representation of endothelial layer of developing vascular wall (*pink*), listed are intra- and extraembryonic sources of vascular endothelial cells with hemogenic potential (*blue box*). **b** Schematic representation of progression of events and molecular signals governing hemogenic specification (*orange boxes*) and **c** endothelial to hematopoietic transition (*green* *boxes*), ending with generation of intra-vascular hematopoietic clusters and multi-lineage hematopoietic stem and progenitor cells (HSPC)

Despite these knowledge gains about the characteristics of hemogenic endothelium over the last decade, little is known about the hierarchy of molecular events that govern their specification from non-blood forming endothelium, as well as the events that lead to their generation of HSPC. However, this body of knowledge is growing and recent advances in our understanding are discussed below (and are summarized in Fig. 1).

### Retinoic acid signaling

Retinoic acid (RA) is known to regulate murine endothelial cell development and specification [73, 74]. Biologically active retinoic acid, known as all-trans retinoic acid (ATRA), is derived from retinol (Vitamin A) via oxidation by retinaldehyde dehydrogenases (RALDH1-3). ATRA is then released, taken up by target cells, and bound to members of the retinoic acid receptor (RAR) family (α, β, or γ). Heterodimerization of RAR family members with rexinoid receptors (RXR) α, β, and γ form active transcription factor complexes that bind to retinoic acid response elements within target genes to initiate transcription (reviewed in Marcelo[1] and Chanda [75]).

During vasculogenesis in the murine yolk sac, active RA is generated by RALDH2 expressed in the visceral endoderm, which then signals within endothelial cells in the adjacent mesoderm that selectively express RAR α-1 and α-2 [73]. *Raldh2*−/− mutant embryos exhibit abnormal vascular development, due to hyperproliferative endothelial cells [74], as well as anemia, and die around E10.0 [76]. Other work has demonstrated that mice deficient for RA receptors have impaired fetal hepatic erythropoiesis [77] as well as impaired generation of marrow-derived HSC [78]. Because of its essential role in endothelial cell development, as well as embryonic and postnatal hematopoiesis, RA signaling is of great interest in the study of specification of endothelial cells to a hemogenic state.

Although *Raldh1* and *Raldh3* null embryos have not been shown to exhibit hematologic defects [79], embryos lacking *Raldh2* were shown to exhibit defects in definitive, but not primitive hematopoiesis [13, 75]. In addition, we found that the underlying hematopoietic defect in *Raldh2−/−* mutants is lack of hemogenic endothelial cell development, which could be rescued via provision of bioactive RA to *Raldh2−/−* embryos in utero or in culture [13, 61]. RA signaling is critical for the development of hemogenic endothelial cells within the yolk sac, as well as AGM. In both tissues, ~90 % of endothelial cells with active RA signaling exhibit a hemogenic phenotype; in addition, ~90 % of hemogenic endothelial cells are undergoing active RA signaling during definitive hematopoiesis in these tissues [61, 75]. These findings place RA at or very near the top of the signaling hierarchy governing hemogenic endothelial cell specification and function during definitive hematopoiesis [13].

### Notch

The Notch signaling pathway is evolutionarily conserved and involved in many aspects of embryonic development, with notable roles in vascular and cardiac development. Mammals express four members of the Notch family of receptors (Notch1-4) and five ligands: Delta-like 1, 3, 4 (DLL) and Jagged 1 and 2 (Jag). The interactions between the receptors and ligands allow signal transduction between neighboring cells via a multistep proteolytic cleavage of the Notch receptor and subsequent release of the Notch intracellular domain (NICD) (reviewed in High 2008 [80]). A γ-secretase complex powers the final step of release of NICD into the cytoplasm, wherein NICD can then enter the nucleus to form active transcriptional complexes with RBPJ, and co-activator Mastermind-like (MAML) [80]. The pathway itself appears straightforward, in theory, but is regulated by several positive and negative feedback loops whose actions are determined by both cellular context, developmental timing, and local environment [80].

Notch signaling is well known to be involved in cell fate decisions and cell differentiation, particularly in endothelial and blood cells, and a growing body of evidence also points toward its involvement in hemogenic specification. Embryos lacking Notch1 or Notch1 and Notch4 receptors, the only receptors expressed by endothelial cells [81, 82], exhibit abnormal vascular development similar to *Raldh 2*−*/*− mutants [74, 83, 84], and the expression of Notch1, and its downstream effectors HES1 and HEY1, which are known to regulate hematopoiesis [85] are upregulated in endothelial cells by RA [61]. In addition, chemical inhibition of Notch signaling in E8.0 wild-type mouse embryos via use of γ-secretase inhibitor DAPT suppresses yolk sac hemogenic endothelial cell specification [61]. Notch1, specifically, is expressed in the ventral wall of the dorsal aorta where hemogenic endothelium forms (reviewed in Jang 2015 [86]), and the AGM of *Notch1*−*/*− mutants exhibit decreased hematopoietic activity (reviewed in Zape 2011 [37, 87]). Conversely, Notch1 induction in murine embryonic stem cells leads to an expansion of VE-cad+ hemogenic endothelial cells with enhanced hematopoietic potential [86]. These data collectively suggest that Notch1 receptor signaling may regulate hemogenic endothelial cell specification and function, and also place Notch downstream of RA in the signaling hierarchy governing hemogenic specification.

### c-Kit

c-Kit (CD117) is a growth factor receptor with tyrosine kinase activity known to bind stem cell factor (SCF), which leads to dimerization of receptors and subsequent activation or regulation of kinase activity that modulates intracellular signal transduction pathways involved in cellular proliferation, maintenance and migration [1]. Such pathways include Src kinase, PI3K, JAK-STAT, MAPK, and PLCγ, as well as regulatory PTPases, phosphatidylinositol phosphatases, and protein kinase C [88]. SCF is known to support multilineage hematopoietic development [88] and c-Kit mutations are associated with embryonic lethality at midgestation, anemia and disrupted HSC development [88, 89]. As previously noted, the expression of c-Kit is a distinguishing feature of hemogenic endothelial cells, relative to non-blood forming endothelial cells. In addition, Notch1 expression in endothelial cells is upregulated downstream of c-Kit; thus, this pathway appears to play a critical, yet still undefined role, in the signaling hierarchy that governs hemogenic endothelial cell specification [61].

### Cell cycle control/p27

Retinoic acid-deficient and Notch-inhibited embryos similarly exhibit endothelial cell hyper-proliferation, and impaired hemogenic endothelial cell development. We found that downregulation of cell cycle inhibitor p27, which induces G1 arrest [73, 74], to be the underlying defect in RA-deficient embryos. Furthermore, p27 is upregulated downstream of Notch and c-Kit, and lentivirus mediated re-expression of p27 in both RA- and Notch-inhibited murine yolk sac endothelial cells rescues endothelial cell cycle control, hemogenic specification, and the generation of HSPC [61]. Given that cell cycle state and duration of G1 phase have been shown to be determinants of stem cell fate [90–92], it is possible that endothelial cells must be arrested in G1 phase in order to undergo hemogenic specification. In fact, several transcriptional regulators of definitive hematopoiesis are differentially expressed in G1 phase (reviewed extensively in [93]). It is also possible that p27 functions as a transcriptional regulator to directly alter endothelial cell phenotype, given that it can repress multipotency genes in stem cell populations [94]. Thus, the RA/c-Kit/Notch signaling axis which mediates p27 expression may regulate hemogenic specification in at least two ways: (1) via repression of genes that maintain a multipotent state in primordial endothelial cells or induction of hemogenic genes; and/or (2) elongation of G1 phase of cell cycle to enable expression and accumulation of transcriptional regulators that alter endothelial cell phenotype [61].

## Generation of hematopoietic stem/progenitor cells from hemogenic endothelium

The process by which HSC are generated from hemogenic endothelium is referred to as the endothelial-to-hematopoietic transition (EHT; depicted in Fig. 1). In order to be able to derive HSC from pluripotent stem cells for clinical and research applications, an understanding of the events that lead to HSC formation from endothelial cells in vivo is required. The events leading to HSC generation from an endothelial intermediate at multiple anatomic sites, including the yolk sac, placenta, and AGM, involves an incompletely characterized complex interplay between microenvironment, developmental timing, and transcriptional regulation of fate.

### The AGM as a source of HSC

Prior to the onset of circulation, it has been shown that lymphocyte and myeloid progenitors emerge as early as E7.5 within the para-aortic splanchnopleura (pSp) [6] and that explants of pSp demonstrate long-term hematopoietic reconstitution ability whereas yolk sac explants demonstrate only short term myeloid reconstitution ability [7]. Similar results were found in *Xenopus* studies that demonstrated HSC emergence from dorsal aortic tissue that derived from a blastomere independent of the yolk sac [95]. The implication of this is that long-term repopulating stem cells, or HSC, are intra-embryonic in origin. Work by several groups led to the discovery of intra-aortic hematopoietic clusters (IAHC) arising from the endothelial lining of ventral wall of the dorsal aorta prior to their release into circulation and eventual long-term colonization of fetal liver and marrow, thus corroborating the intra-embryonic origin of HSC [43, 53, 55, 57, 96–101]. IAHC have been shown to appear around E9.5 in the AGM, and peak emergence of HSC from them occurs between E10.5 and E11.5 [21–23, 96].

The cellular composition and function of IAHC before and during HSC detection has been investigated, although to date a unified cell surface marker profile of HSC generated within the embryo has not been identified, presenting an important roadblock to furthering our understanding of EHT. IAHC at E10.0 were found to be phenotypically heterogeneous and contain very few progenitors (average of 22), but notably contain pre-HSC (average of 12) capable of long-term multilineage hematopoietic reconstitution post-transplantation [102]. A collection of hematopoietic, stem, and endothelial markers such as c-Kit, CD31, CD34, SCA-1, MAC1, VE-cadherin, Tie2, Flk-1 and CD45 have been shown to be expressed by HSC within the embryo, but these are also found on other cell types [47, 57, 99, 103–106]. Intra-aortic hematopoietic clusters have also been shown to be composed of pre-HSC that differentially express endothelial and hematopoietic surface markers including VE-cadherin, c-Kit, Ly6a, CD41 and CD45, suggesting that a post-hemogenic endothelial cell intermediate may exist along the transition from endothelial cell to HSPC within these intra-aortic clusters [53, 102, 106–108]. The remaining cell types within IAHC are yet to be identified, but are hypothesized to be comprised of these immature pre-HSC that will mature toward a HSC fate via EHT. Once the hematopoietic cluster phase of EHT is complete, data from chick studies show that the splanchnopleural endothelium in the aortic floor is replaced by somitic endothelial cells, in part offering an explanation for the very brief timeframe in which vascular hematopoiesis occurs during development [109], although this has not been shown in mouse models [110].

The dorsoventral polarity of IAHC emergence appears to be critical to governing their ultimate hematopoietic fate. This has been shown in the chick [111] and mouse [112] to be guided by local presence of mesenchymally derived pro-hematopoietic ventralizing (VEGF, bFGF, TGF-β, BMP4) or anti-hematopoietic dorsalizing factors (EGF and TGF-α), the downstream effects of each impact the expression of critical hematopoietic transcription factors that are involved in EHT [14, 111, 112]. Importantly, sub-aortic mesenchyme has not been shown to be a direct source of hematopoietic cells [45], thus corroborating the importance of these locally derived factors in driving hematopoietic specification of vascular endothelial cells. HSC have been found to have particular enrichment in the middle third of the E11.0 dorsal aorta, immediately adjacent to origin of the vitelline artery, suggesting that a disturbance in flow at the junction of these two vessels may interact with microenvironmental signals to drive HSC emergence [113]. The presence of hematopoietic mediators such as RUNX1 in the ventral aortic mesenchyme [56, 57], and upregulation of genes involved in cell death, adhesion, migration, as well as vascular development and hematopoiesis [113], coupled with restriction of definitive HSC localization to the ventral aorta [99] and evidence demonstrating that para/peri-aortic supporting cells also promote hematopoiesis [111, 112, 114–116], suggests that within the AGM there likely exists a specialized hematopoietic microenvironment or niche [117] powered by master transcriptional regulators, their downstream effectors, and extrinsic modifiers of these signaling cascades such as fluid shear stress [118, 119] that promote initial HSC development from hemogenic endothelium—these are reviewed below.

### RUNX1

RUNX1 (AML1) is a sequence-specific DNA binding protein that is part of a family of transcription factors called core binding factors (CBF), and an essential master regulator of EHT during aortic hematopoiesis [58, 120–122]. Consistent with a role in EHT, RUNX1 deficiency in embryonic stem cells prevents the formation of blood cells from hemogenic endothelium [42]. It is expressed in all sites of definitive hematopoiesis in the embryo and precedes emergence of HSPC. Interestingly, this is conserved across all vertebrate species studied [123]. RUNX1 is expressed by mesenchymal cells in the dorsal aorta and AGM, placenta, and in hematopoietic cell clusters in ventral-dorsal aorta and vitelline and umbilical arteries [56, 57, 123], but its requirement, while critical for normal definitive hematopoietic development [124–126], as previously reviewed [14], is transient in embryonic development, and is not required during primitive hematopoiesis [42, 121] or after EHT [58]. RUNX1 expression has been shown to repress the endothelial program, while activating the hematopoietic program, as evidenced by sequential emergence of hematopoietic markers CD41 and later CD45 [42, 106, 108], at the time of transition from hemogenic endothelial cells to a hematopoietic cell fate [120, 123]. Deletion of *Runx1* does not prevent emergence of the CD41 marker on VE-cad+CD45-cells, but has been shown to prevent transition of these cells to CD41+CD45+ [127]. This patterned transition from endothelial cell phenotype to a hematopoietic phenotype is in part controlled by binding of multiprotein complexes containing GATA, Ets (PU.1) [128], and SCL family factors to *Runx1* enhancers known to be involved in HSC emergence [14, 129, 130] and is also marked by loss of expression of genes associated with arterial identity, such as *Sox17* and *Notch1* [131], further supporting the idea of temporal transcriptional regulation governing cell fate as a central tenet of EHT.

Prospective isolation and transcriptional analysis of murine hemogenic endothelial cells using a transgenic *Runx1* enhancer-GFP reporter model has demonstrated that beginning at E9.5, hemogenic endothelial cells commit to hematopoietic lineage in an ordered fashion marked by an early gradual loss of endothelial potential and increase in hematopoietic potential. This occurs while the hemogenic endothelial cells still remain part of the endothelial wall [59]. The different stages of the transition from endothelial to hematopoietic cell have also been shown to be guided by the transcriptional activity of *Runx1*'s two [132] promoter regions: the P1 (distal) and P2 (proximal) *Runx1* promoters control expression of *Runx1c* (distal) and *Runx1b* (proximal) isoforms [133, 134]. RUNX1b is expressed at low levels in hemogenic endothelium until E10.5 [135, 136], whereas RUNX1c expression is initiated within HSPC and loss of endothelial phenotype [133, 134, 136]. The P1 promoter element associated with RUNX1c expression is more complex than the P2 promoter and contains many binding sites for hematopoietic transcription factors [132]. RUNX1b transcriptome analysis reveals that it activates genes associated with cell adhesion, ECM remodeling, integrin signaling, and endothelial cell migration at the onset of transition from hemogenic endothelial cells to HSPC [133]. RUNX1 downstream targets such as GFI1/GFI1B are also thought to promote down-regulation of endothelial proteins, thereby promoting a morphologic change from flattened endothelial cells to round cells [137]. Lack of GFI1B in embryos results in failure of release of hematopoietic cells from the extraembryonic yolk sac vasculature into circulation [137]. Generation of P1 and P2 RUNX1-EGFP zebrafish reporter lines demonstrated erythromyeloid progenitor emergence from the posterior blood island at 18hpf in the P1 line and definitive HSC emergence in the AGM region at 22hpf in the P2 line, further underlining the role of alternative *Runx1* promoter usage in cell fate determination via generation of spatially segregated yet temporally successive HSPC emergence [138]. Other work further demonstrated that the RUNX1c isoform was indeed only expressed at the time of emergence of definitive HSC (E10.5-11.5) in the mouse AGM and human (embryoid body day 12), but retroviral overexpression of both RUNX1 isoforms in mouse marrow and liver HSPC demonstrated no functional difference between either isoform in further generation of HSC, and induced quiescence in mouse HSC in vitro and in vivo. This suggests a developmentally important timeframe for the RUNX1c isoform in specification of HSPC from hemogenic endothelial cells at the time of EHT [139]. Other studies have identified a role for blood flow induced shear stress as a regulator of Runx1 expression by the endothelium [118, 119].

Studies utilizing conditional deletion of *Runx1*, driven by *Vav1* promoter expression, a pan-hematopoietic gene, demonstrate that its hematopoietic requirement only extends through the transition from endothelium to HSPC [58]. Dependence on RUNX1 for generation of erythromyeloid progenitor cells begins at E7.5 [140] and ends around E10.5 [141]. Further study of requirement for RUNX1 in hematopoietic development utilized timed endothelial-specific deletion of RUNX1 activity showed that the transition to RUNX1 independent HSC formation occurs around E11.5 and does not require fetal liver colonization [141]. Tissue-specific restoration of RUNX1 function in murine Tie-2-expressing cells allowed for rescue of embryonic definitive HSC potential, allowing mutants to survive until birth without the previously described hemorrhagic phenotype observed in *Runx1* null embryos. These observations suggest that the primary embryonic lethal defect in *Runx1* mutants is indeed hematovascular in nature [142]. In adults, deletion of *Runx1* does not cause a reduction in HSPC numbers in the marrow; in fact, it causes an expansion of these cell types [123] and leads to hematologic abnormalities, such as increased myeloproliferation with neutrophilia, thrombocytopenia, and increased extramedullary hematopoiesis [143]. In the absence of RUNX1 during development, primitive erythropoiesis proceeds normally, hemogenic endothelium develops, but neither generation of myeloid and lymphoid progenitors, nor HSC generation occurs (reviewed in [14]), leading to an absence of IAHC and fetal liver HSC [54, 56, 57, 144], and ultimately death by E12.5 [144]. Interestingly, studies of hematopoietic tissues from *Runx1* haploinsufficient embryos yields variable degrees of HSC generation depending on the tissue type isolated, such that cultured mutant AGM explants yield fewer HSC than wild-type, and yolk sac and placental tissues yield increased numbers of HSC [145]. On the other hand, AGM-derived HSC from *Runx1* haploinsufficient mice that are directly transplanted into irradiated recipients without an intermediate culture step demonstrates increased generation of HSC [57]. Collectively, this body of evidence strongly supports a role for RUNX1 as a critical regulator of EHT, and that modification of its expression via interaction with associating proteins within specific tissue microenvironments and developmental time points, as well as under different physiologic conditions, impacts regulation of the hematopoietic transition [14, 124, 145].

### SCL/LMO/GATA

The regulatory regions of hematopoietic genes are known to be bound by RUNX1, as discussed, as well as a transcription factor complex composed of stem cell leukemia protein (SCL)/T cell acute lymphocytic leukemia protein-1 (TAL-1), LIM domain only 2 (LMO2), GATA1, and GATA2 ([1, 140, 146–148], all of which have been implicated in EHT. LMO2 is thought to act as a bridge between SCL/TAL-1 and GATA-binding proteins in a transcriptional activating complex that drives vertebrate hematopoietic specification (reviewed in [1]).

Studies of *Scl/Tal*-*1*, *Lmo2,* and *Gata1/2* mutant mouse embryos demonstrate that deletion of each gene results in midgestation lethality and impaired definitive hematopoiesis [149–152]. SCL is a helix-loop-helix transcription factor that functions upstream of RUNX1, and has been shown to be a critical regulator of hematopoiesis via generation of Tie-2hi c-Kit+CD41− hemogenic endothelium [42].

SCL loss-of-function studies show that hematopoiesis is impaired at both the level of stem cell formation and subsequent differentiation [153]. Interestingly, multiple isoforms of SCL [SCLA (full length) and SCLB (N-terminal truncated)] have been identified [153], and may play differential roles in definitive hematopoiesis. In vivo time lapse imaging studies demonstrated selective SCLB isoform expression in the dorsal aorta of hemogenic endothelium just prior to EHT, and this isoform may act upstream of RUNX1 to mediate EHT. SCLA, on the other hand, is expressed in HSC after the EHT process, and is critical for maintenance of these newly born cells within the AGM [153].

GATA2 is expressed by the para-aortic splanchnopleura and AGM regions of the mouse embryo [154]. It has been shown to have a role in production and expansion of HSC in the AGM during embryogenesis as well as normal expansion of the adult HSC pool within the bone marrow [155]. *Gata2 *deficient mice die at E10.5 due to defective primitive erythropoiesis and lack of HSPC generation [151]. Conditional deletion of a *Gata2* cis regulatory element in mouse AGM results in reduced expression of hematopoietic transcription factors SCL and RUNX1, and hemogenic endothelial cells of the mutants fail to generate HSPC, leading to embryonic lethality by E13-14 [156]. Additionally, VE-cadherin-driven deletion of *Gata2* prevents generation of intra-aortic clusters in mouse dorsal aorta and results in a deficiency of long-term repopulating HSC [157]. Embryonic stem cells derived from adult chimeric mice deficient in *LMO2* also fail to contribute to the endothelium of large vessels [158, 159] and blood cell production [158, 159]. Morpholino knockdown of either *Scl* [147] or *Lmo2* [160] in zebrafish results in similarly impaired expression of hematopoietic genes and loss of IAHC. Collectively, these findings implicate this group of transcription factors as having essential roles in EHT and definitive hematopoiesis.

### Notch

In addition to the previously discussed role for Notch signaling in hemogenic specification, Notch has also been shown in a number of vertebrate studies to be essential for definitive HSC emergence in the embryo [87, 161, 162]. It is thought that Notch signaling mediates HSC development via EHT by interactions between emerging HSPC expressing Notch receptors and underlying endothelial cells and stroma expressing Notch ligands [163, 164]. Notch1 and Notch4 receptors are expressed specifically in the endothelial cell layer of the dorsal aorta, and Notch signaling pathway components such as DLL4, Jag1, Jag2, HES1, HRT1, HRT2, and GATA2 are expressed in both the endothelial cell layer of the dorsal aorta and its emerging hematopoietic clusters beginning between E9.5 and E10.5 [165–167]. Mouse and zebrafish embryos that lack Notch signaling components demonstrate defective or absent definitive intra-embryonic hematopoiesis and inability to establish permanent self-renewing HSC (reviewed in [1, 165]). In-vitro blockage of Notch signaling using DAPT has been shown to prevent EHT of cultured murine E9.5 pSP/AGM cells [163]. Activation of Notch signaling in a modified culture system of E11.0 pSp/AGM hemogenic endothelial cells on immobilized chimeric human DLL1 results in emergence of multilineage hematopoietic progenitor formation with long-term engraftment capability [163], further supporting the critical role of Notch in driving early hematopoiesis.

HSC generation in the hematopoietic clusters of the murine AGM has been shown to mostly depend on Notch ligand Jag1 and its regulation of *Gata2* expression [167]. The *Gata2* promoter region contains two RBPJ binding sites involved in Notch-mediated activation [168] and not surprisingly, deletion of Notch transcriptional co-activator RBPJ leads to loss of HSPC, as well as loss of GATA2 expression, which is necessary for continued differentiation of hematopoietic cells [166, 169]. Notch-dependent GATA2b expression has been demonstrated to be required for RUNX1 expression within zebrafish dorsal aorta from a very early developmental stage, and its expression is limited to emerging HSC [170]. Embryos deficient for Notch1 downstream targets HES1 and HES5 have been shown to have intact arterial specification but have notable hematopoietic defects marked by abnormal proliferation of nonfunctional HSPC and increased expression of hematopoietic regulators including GATA2, RUNX1, and c-Myb [168]. Further interrogation of the *Gata2* promoter region in this study revealed that in addition to containing RBPJ binding sites essential for initiation of hematopoiesis, it also contained HES binding sites necessary for *Gata2* downregulation involved in driving functional hematopoiesis [168]. This highlights the complexity of Notch signaling in this context, as both driving expression of and concomitantly negatively regulating a key hematopoietic mediator.

Previous work showing that different Notch ligands confer different signal strengths [171, 172] has formed the basis for recent work suggesting that determination of endothelial versus hematopoietic fate in the developing AGM is mediated by ligand-specific differences in Notch1 signal strength. Use of high and low sensitivity Notch1 activation trap mouse models demonstrated that hematopoietic specification and repression of the endothelial program is driven by low levels of Jag1 mediated Notch signaling. In the absence of Jag1, DLL4 mediated high levels of Notch activity instead drives the endothelial/arterial program [173]. The authors of this study propose a model wherein Jag1 antagonizes DLL4-mediated high Notch signaling in a subset of endothelial cells to drive them toward a hematopoietic fate rather than an endothelial fate [173]. This further highlights the critical role of Jag1, as previously discussed, in driving EHT and importantly also adds to the body of evidence that endothelial and hematopoietic cells represent distinct lineages.

In human embryonic stem cells, Notch activation has also been demonstrated to be necessary for generation of CD45 + cells, and Notch signaling via HES1 is necessary for their hematopoietic differentiation [174]. Other studies in human embryonic stem cells showed that ligand DLL4 is induced via Notch signaling in a small subset of endothelial progenitors during hematopoietic differentiation, and the level of DLL4 expression appears to correlate with hematopoietic versus endothelial fate. That is, DLL4-high progenitors are enriched for endothelial potential, whereas DLL4low/− coincides with acquisition of hematopoietic potential [174]. Thus, a model has been proposed, wherein hemogenic endothelial cells are DLL4low/− and are Notch activated via neighboring DLL-high cells to promote transition from an endothelial state to CD45+ hematopoietic cells that cluster in a fashion similar to intra-aortic hematopoietic clusters in the AGM [174]. Related studies also demonstrated a functional role for DLL4 in promoting specification of hematopoietic cells from hematoendothelial progenitors, wherein expression of DLL4 increased numbers of clonogenic hematopoietic progenitors, and interestingly skewed their fate toward an erythroid lineage [174]. No correlation between levels of Jag1 or Jag2 expression was noted with respect to generation of hematoendothelial progenitors or hematopoietic precursors there from, as has been noted in other vertebrate studies, suggesting that human HSC differentiation may be affected by other Notch ligands [174].

A recent study investigated Notch-regulated elements involved in control of HSC generation in zebrafish, mouse, and human embryonic tissues. ChIP on ChIP analysis against Notch co-activator RBPJ was performed and identified candidate promoter regions of genes regulated by Notch in hemaotpoietic tissues. The most significant result was enrichment of *Cdca7*, which is shown to recruit RBPJ, as well as Notch1 ICD. CDCA7 expression was shown to be upregulated in the hemogenic population derived from human embryonic stem cells in a Notch-dependent manner. Down-regulation of *Cdca7* mRNA was shown to induce hematopoietic differentiation and concomitantly decrease HSPC numbers, suggesting that it is a Notch-mediated target involved in emergence of HSPC, but not differentiation of these cells [175].

### Wnt/β-catenin

There is evidence that Notch, Wnt, and BMP pathways interact to generate HSC in the zebrafish embryo and also are involved in driving hematopoietic development from embryonic stem cells [176, 177]. Wnt is a well-described evolutionarily conserved signaling pathway known to modulate many areas of embryonic development. The Wnt ligands are a family of 19 glycosylated proteins that bind Frizzled receptors and LDL receptor protein co-receptors to trigger a variety of downstream responses including activation of β-catenin (canonical pathway) and/or JNK and PKC (noncanonical pathway) that modulate gene expression programs critical for normal embryonic development (reviewed in [176]). Early evidence that Wnt/β-catenin has a role in embryonic hematopoiesis came from zebrafish studies showing Prostaglandin E2 mediates HSC formation in the AGM via β-catenin [177]. Recently, it has been shown that EMP cells emerging from vascular endothelium in the yolk sac do so independent of vascular identity or the influence of circulation, but their emergence requires intact Wnt signaling [12]. It has been shown that Wnt/β-catenin activity is transiently required in the AGM of mouse embryos for emergence and generation of long-term HSC, as well as production of hematopoietic cells in vitro from AGM endothelial precursors [176]. Hemogenic endothelial cells from AGM at E11.5 have also been shown to have a high degree of active Wnt signaling, as evidenced by the presence of high levels of β-catenin in cells lining the dorsal aorta, close to emerging hematopoietic clusters, but not in cells budding off of endothelium or within circulating plasma [75]. In human pluripotent stem cell models of EHT, hemogenic endothelium undergoes EHT toward either a primitive or definitive hematopoietic fate depending on presence of either inactive (primitive) or active (definitive) Wnt signaling [178]. This body of evidence suggests a context-specific role for Wnt such that it is required for the generation of hemogenic endothelium and likely initiation of EHT but gradually becomes downregulated as HSPC are generated [75].

### HOX/SOX

Homeobox genes, such as *Hox* and *Sox* genes, play a key role in determining cell identity during embryonic development [179–182]. *Hox* paralogue group 3 (HOXA3), specifically, has an important role in endothelial and cardiovascular development (reviewed in [183]). Its expression was found to be high in embryonic mesenchymal tissue, intermediate in the dorsal aorta and absent from the yolk sac at E8.25 and E8.5 when definitive hematopoiesis is occurring in that tissue. This is consistent with the idea that it prevents hematopoietic differentiation of endothelial progenitors via down-regulation of RUNX1, GATA1, GFI1b, Ikaros and PU.1. Conversely, downregulation of HOXA3 is concomitant with increased RUNX1 expression within the aortic endothelium, and its re-expression in embryonic stem cells, as well as cultured mouse embryonic tissues, causes retention of an endothelial phenotype. These data suggest an important role for HOXA3 in maintenance of an endothelial state prior to hematopoietic transition [183]. Ectopic expression of another *Hox* paralogue, HOXB4, in yolk sac derived hematopoietic progenitor cells has been shown to drive conversion to a definitive HSC phenotype with long-term engraftment potential [184]. Additionally, several transcriptional regulators of the definitive hematopoietic program such as RUNX1, SCL/TAL1, GATA2, and GFI1 have been shown to be direct targets of HOXB4 in embryonic stem cell-derived hematopoietic progenitors [185].

SOX17 is a transcription factor expressed in AGM hemogenic endothelium downstream of HOXA3 [183] at E8.5–8.75, and in addition to its role as a critical factor governing arterial identity [186], it has been shown to be required for generation of fetal and neonatal HSC [187]. Although SOX17 is not required for the generation of adult HSC [120], its overexpression in adult hematopoietic progenitors has been shown to confer fetal HSC characteristics [188]. Additionally, SOX17 can drive expansion of cells that exhibit downregulation of endothelial markers and also possess an ability to generate hematopoietic cells [120, 187, 189]. Loss of *Sox17* decreases expression of Notch1 in the murine AGM, suggesting that SOX17 mediates generation of hemogenic endothelium via Notch signaling during definitive hematopoiesis [187]. Conditional loss of SOX17 mediated repression of *Runx1* and *Gata2* recently has been shown to result in increased production of hematopoietic cells from murine AGM endothelial cells that can be dampened by an increase in Notch signaling [131], suggesting that following hemogenic specification, SOX17 modulates hemogenic endothelium by active repression of the hematopoietic program by the SOX17/Notch axis until initiation of EHT. This has been complemented by studies of murine AGM hemogenic endothelial cells undergoing EHT using combined correlative scanning electron microscopy and immunofluorescence demonstrating an increase in RUNX1 levels concomitant with decrease in nuclear SOX17, rounding of previously flattened AGM endothelial cells, and the onset of co-expression of CD41 and c-Kit on these newly rounded cells [190]. SOX17 has also recently been shown to be necessary for commitment to the definitive erythroid lineage [191]. Other closely related SOX family members, SOX7 and SOX18, have a role in primitive hematopoiesis. SOX7 and SOX18 have transient expression in hematopoietic precursors at the onset of blood specification and expression of these in early hematopoietic precursors from mouse embryonic stem cells and embryos enhances their proliferation while blocking their maturation [189, 192, 193].

### cMyb

The transcription factor cMyb was originally identified as a nuclear protooncogene involved in certain avian cancers but is also expressed in HSPC prior to their differentiation. Multiple mouse studies utilizing *cMyb* mutants demonstrate the key role played by cMyb within a variety of hematopoietic pathways such as lymphoid development, HSPC maintenance, and HSPC differentiation [194]. Deletion of *cMyb* results in murine embryonic lethality at E15.5 due to lack of fetal liver hematopoiesis, specifically impaired erythroid and myeloid development [195]. AGM explants from *cMyb*–/– mutant mice also yield 100-fold fewer HSPC [196]. Loss of function studies in zebrafish have shown similarly impaired definitive hematopoiesis, even in the setting of otherwise normal primitive hematopoiesis [197, 198]. Zebrafish studies have also demonstrated accumulation of HSPC within the ventral wall of the dorsal aorta, their site of origination, and that this failure of migration to a hematopoietic niche such as the zebrafish caudal hematopoietic tissue and kidney may in part underlie the previously demonstrated failure of definitive hematopoiesis in *cMyb* mutants [194].

## Other mediators of HSPC generation from hemogenic endothelium

### G-protein coupled receptors

To identify candidate regulators of EHT on a global level, whole genome transcriptome analysis of murine aortic endothelial cells, hemogenic endothelial cells and HSPC populations has identified 530 differentially expressed genes during EHT with particular upregulation of the gene encoding G protein coupled receptor 56 (*Gpr56*) and heptad transcription factors SCL, LYL1, LMO2, GATA2, RUNX1, ERG, and FLI-1) [199, 200]. The heptad factors were been shown to bind to *Gpr56* enhancer regions and regulate its expression, and knockdown of *Gpr56* in zebrafish resulted in hematopoietic defects that could be rescued with either mouse or zebrafish *Gpr56* RNA, thus establishing GPR56 as a novel regulator of EHT via a yet to be elucidated mechanism [199]. Another G protein coupled receptor, GPR183, has also recently been shown to dampen Notch signaling in zebrafish models via its recruitment of β-Arrestin-1 and E3 ligase NEDD4, both of which degrade Notch1 in hemogenic endothelial cells to promote EHT [201].

### Purine signaling

Purines (such as adenosine, ADP, and ATP) exhibit extracellular signaling activity that regulates diverse physiologic and developmental cellular functions including autoregulation of blood flow, cell proliferation and differentiation, and stem cell regeneration, all via cell surface receptors [202]. Adenosine signaling at the level of the vascular endothelium has recently been demonstrated to play a role in regulation of HSPC development in both zebrafish and mice. In a recent study, elevated adenosine levels in zebrafish embryos were shown increase numbers of RUNX1+/cMyb+ HSPC in the dorsal aorta via increased expression of CXCL8 (IL-8), which has been shown to trigger proliferation of hematopoietic progenitor cells [203]. Furthermore, in the same study, alteration of adenosine signaling via knockdown of the A_2b_ adenosine receptor resulted in disruption of both the generation of SCL+ hemogenic vascular endothelium and the endothelial to hematopoietic transition. These results were mirrored in mouse embryonic stem cell colony forming assays, as well as E10.5 AGM explants, that demonstrated an increase in production of multipotent progenitor colonies in the setting of increased adenosine signaling [202].

### Chromatin remodeling

The role of reprogramming of transcriptional states in promoting lineage commitment in HSC continues to be of great interest in the field. The role of chromatin modification as an important mediator of transcriptional regulation of EHT has been investigated. Majumder and coworkers demonstrated that the histone chaperone HIRA (histone cell cycle regulation-defective homolog A) regulates RUNX1 activity during EHT. They showed that HIRA mediated histone acetylation of *Runx1* activates the downstream hematopoietic targets of RUNX1 and that in *HIRA* deficient mutants, there was not only reduced expression of *Runx1*, but also lack of chromatin reorganization necessary for appropriate RUNX1 binding and transcriptional activity to drive hematopoiesis [204]. Chromodomain helicase DNA-binding protein 1 (CHD1), an ATP-dependent chromatin-remodeling enzyme whose activation is associated with an increased level of total active transcription in mouse embryonic stem cells [205, 206] has also recently been shown to be essential for successful EHT. Endothelial-specific *Chd1* mutants, while able to produce intact E10.5 IAHC, that show intermediate markers of differentiation such as RUNX1 and c-Kit, do not successfully initiate definitive hematopoiesis and die by E15.5 due to severe anemia and absolute failure of erythropoiesis [207]. Analysis of cells from the mutant IAHC demonstrated that the cells in the clusters underwent apoptosis rather than hematopoietic specification, as evidenced by failure of expression of markers of hematopoietic lineage such as CD45, and a fivefold reduction in myeloid colony forming potential. Global gene expression analysis of *Chd1* mutant endothelium at E10.5 in this study was generally found to be unchanged when compared to wild-type endothelial controls, but notably lacked activation of a set of 156 genes implicated in hematopoiesis and growth. Furthermore, hematopoietic progenitors underwent a CHD-1 dependent elevation in global transcriptional at the time of EHT that was necessary for initial expansion and survival of HSPC but not for differentiation and maintenance of the differentiated HSC pool [207]. Committed hematopoietic progenitors with a *Vav*-Cre *Chd-1* mutation demonstrated peripheral blood composition comparable to wild type mice at one year of age. Taken together, these results suggest a pointed critical role for CHD-1 mediated increased transcriptional output in order to successfully complete EHT and establish functional hematopoiesis, but that it is not required beyond the point of hematopoietic specification [207].

### Hypoxia and inflammation

The intrauterine environment is notably hypoxic and via the action of hypoxia inducible factor transcription factors (HIF), transcription of genes necessary for promoting normal development in this environment is activated (reviewed in [208]). The midgestation AGM and placenta have been shown to exhibit localized areas of hypoxia including hematopoietic clusters and some underlying endothelial cells. The role of HIF1α in regulation of embryonic hematopoiesis was recently shown to be necessary for generation of HSPC from the AGM from E9.0 to E11 [208].

Recently, an interesting link between Toll-like receptor 4 (TLR4) inflammatory signaling, a cascade previously associated with stress-induced hematopoiesis in adult marrow, and embryonic hematopoiesis has also been established. TLR4-NF-κB signaling was found to regulate hemogenic endothelium-derived HSPC development in both mice and zebrafish embryos via activation of Notch signaling [209]. This complements other recent studies investigating the role of non-infectious/sterile pro-inflammatory signaling via TNFα or interferon in HSPC development in zebrafish and mice [210–212], suggesting that a low-grade pro-inflammatory state is necessary for normal HSPC emergence even under low-stress conditions and may in fact prime the hematopoietic system for reactive hematopoiesis in the setting of exogenous stressors such as infection [209].

## Summary and conclusions

In summary, the developmental hierarchy governing hematovascular development is complex and multifactorial and our understanding of it is still in its infancy. Over time the study of hematologic development has founded many concepts central to the field of stem cell research as a whole [213]. Hematopoietic development begins early in the mammalian embryo in the extra-embryonic yolk sac and placenta and peaks in the AGM region by E10.5. It is characterized by overlapping waves of generation of primitive blood cells, multilineage hematopoietic progenitor cells, and finally definitive long-term repopulating HSC via a hemogenic endothelial cell intermediate. Emergence of blood cells from hemogenic endothelium is known as the endothelial to hematopoietic transition, which is driven by an incredibly complex and yet to be fully elucidated interplay between developmental timing and tissue microenvironmental factors. This interplay is thought to govern a timed gradual loss of endothelial characteristics [105], accompanied by changes in level and type of expression of hematopoietic regulators such as RUNX1 [56, 99, 142] beginning in the extraembryonic yolk sac, placenta and umbilical vessels, as well as the midgestation aorta, and possibly being completed in hematopoietic niches such as the fetal liver, and bone marrow [18, 19, 99, 102, 105, 106].

Still, there remain a number of unanswered questions surrounding the progression from embryonic mesoderm to endothelium to HSPC and adult HSC. Of particular interest, as already mentioned, is study of the tissue specific microenvironments in which these progenitor cells are generated and mature during development, and the role that microenvironment plays in driving the formation of multilineage HSPC [29]. To date, there is little evidence to support the existence of hemogenic endothelium in later stages of embryonic development or postnatally, but human cord blood hematopoietic progenitors have been shown to differentiate into endothelial precursors capable of generating functional vasculature in vivo and if further instructed by hematopoietic growth factors, first switch to transitional CD144+ CD45+ cells and then to hematopoietic cells perhaps due to the influence of their tissue microenvironment [214]. This finding is of great interest, as it suggests that hemogenic endothelium is not as transient in nature as previously thought and may exist beyond the embryonic period as an untapped reservoir of hematopoietic potential for therapeutic applications [214]. Efficient generation and differentiation of hematopoietic cells in vitro to date has been fraught with technical difficulty and this difficulty is almost certainly exacerbated by a need for a more complete understanding of in vivo endothelial and hematopoietic development (reviewed in [215]).

Further study of these processes, from specification of hemogenic endothelium to generation of HSPC via EHT, and establishment of functional definitive hematopoiesis both in the prenatal and postnatal period, will further our understanding of developmental hematopoiesis, as well as adult hematopoiesis in both health and disease. It will also highlight the cellular and molecular underpinnings of the evolution of a population of stem and progenitor cells that hold great promise for use in clinical therapies for hematologic, oncologic, vascular, and immune-mediated pathologies.
